# Deep learning in renal ultrasound: applications, challenges, and future outlook

**DOI:** 10.3389/fonc.2025.1730628

**Published:** 2026-01-12

**Authors:** Yong Zhang, Yao Hou, Tingting Qiu, Yan Zhuang, Ke Chen, Wenwu Ling, Yan Luo, Jiangli Lin

**Affiliations:** 1 College of Biomedical Engineering, Sichuan University, Chengdu, China; 2 Department of Medical Ultrasound, West China Hospital, Sichuan University, Chengdu, China

**Keywords:** renal ultrasound, deep learning, chronic kidney disease (CKD), multimodal data, large model technology

## Abstract

Kidney disease poses a significant global health burden, often progressing to end-stage renal disease with serious complications. Renal ultrasound, which is real-time, accessible, and noninvasive, serves as a primary imaging tool for evaluating renal structure and pathology. However, its diagnostic accuracy is limited by interobserver variability. Artificial intelligence (AI), particularly deep learning (DL), offers a promising solution for enhancing objectivity and automation throughout the renal ultrasound workflow. This review systematically summarizes DL applications across key tasks—including kidney segmentation, volume measurement, functional prediction, and disease diagnosis—and evaluates the performance of models such as CNNs and transformers. The results indicate that DL has significantly improved the accuracy and efficiency of kidney disease analysis, including chronic kidney disease (CKD), but challenges remain in terms of data quality, model interpretability, generalizations, and clinical integration. In the future, the combination of DL with multimodal data, large model technology, federated learning and interpretable artificial intelligence will be essential to achieve intelligence, standardization and personalization of renal ultrasound.

## Introduction

Renal diseases have become a major challenge in global public health (1). CKD affects more than 850 million people worldwide and is one of the leading causes of death (2). Owing to its advantages of noninvasiveness, real-time imaging, and economy, ultrasound has become the core imaging method for diagnosing and treating renal diseases. As a primary diagnostic method, it can clearly display the structure of the kidneys (size, shape, and cortical thickness) and the state of the collecting system and has high sensitivity for detecting structural abnormalities such as hydronephrosis and renal stones (3–5). Doppler technology can also assess renal vascular hemodynamics and assist in diagnosing functional lesions (6–8). In terms of disease detection, ultrasound can detect various renal lesions, such as congenital abnormalities, stones, cysts, and tumorous lesions (9). In kidney transplant patients, ultrasound is indispensable for evaluating the function and vascular complications of the transplanted kidney (10, 11). New technologies such as ultrasound elastography can also be used to assess the degree of renal fibrosis quantitatively (12). However, traditional ultrasound has significant limitations: (1) it is highly dependent on the operator, and different physicians have low consistency in judging small tumors; (2) it relies mainly on qualitative assessment and lacks objective quantitative indicators such as elastic parameters; and (3) the diagnostic efficiency for complex cases is limited (13, 14).

**Figure 1:**
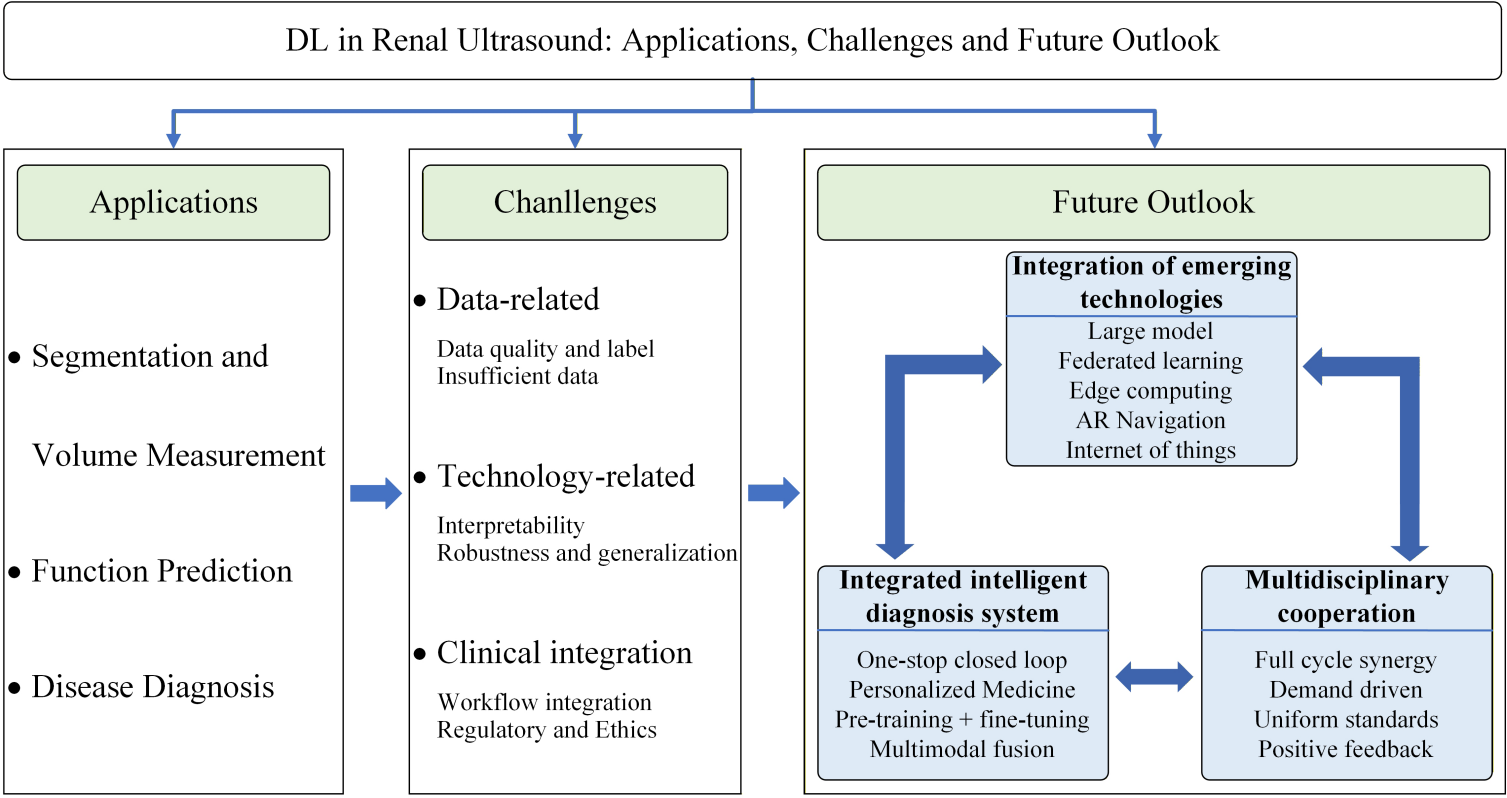
The main framework of this article.

In recent years, AI technology has provided a revolutionary solution to overcome the limitations of traditional diagnosis (15, 16). In the field of renal ultrasound, AI has evolved from traditional machine learning to deep learning, significantly improving the accuracy and efficiency of image analysis. Convolutional neural networks (CNNs) excel in local feature extraction and perform well in kidney image classification and segmentation (17, 18). ResNet solves the problem of gradient disappearance in deep networks through residual connections, improving the accuracy of identifying complex renal boundaries (19). The self-attention mechanism of the transformer model can capture global feature correlations, helping to analyze the spatial relationship between the kidney and surrounding tissues (20). With the optimization of deep learning algorithms, renal ultrasound diagnosis is shifting from an experience-dependent mode to an intelligent and standardized mode, providing a new path for improving the effectiveness of renal disease diagnosis and treatment (21, 22). In recent years, several reviews on AI in the diagnosis and treatment of kidney diseases have been published. For example, De et al. (15) outlined the potential of deep learning in renal ultrasound from the perspective of a technical basis and clinical application, but their discussions are mostly limited to algorithm performance and lack systematic construction of clinical transformation pathways. Although Xu et al. (16) further explored the multitask application of AI in renal ultrasound, their research focused mainly on technical reviews, and no specific technology integration scheme or expansion center collaboration mechanism was proposed. This article not only focuses on the full-chain application of AI in renal ultrasound (from image acquisition to clinical decision support) but also systematically summarizes its application status and frontier progress in kidney segmentation, functional assessment, disease diagnosis and other aspects. It also proposes the construction of a structured framework for clinical transformation for the first time and deeply discusses frontier directions such as multimodal fusion, federated learning and large models. This study provides a systematic reference for promoting the standardized application and precise decision-making of AI technology in renal ultrasound diagnosis. The main framework of this article is shown in **Figure 1**.

## Methodology

**Figure 2:**
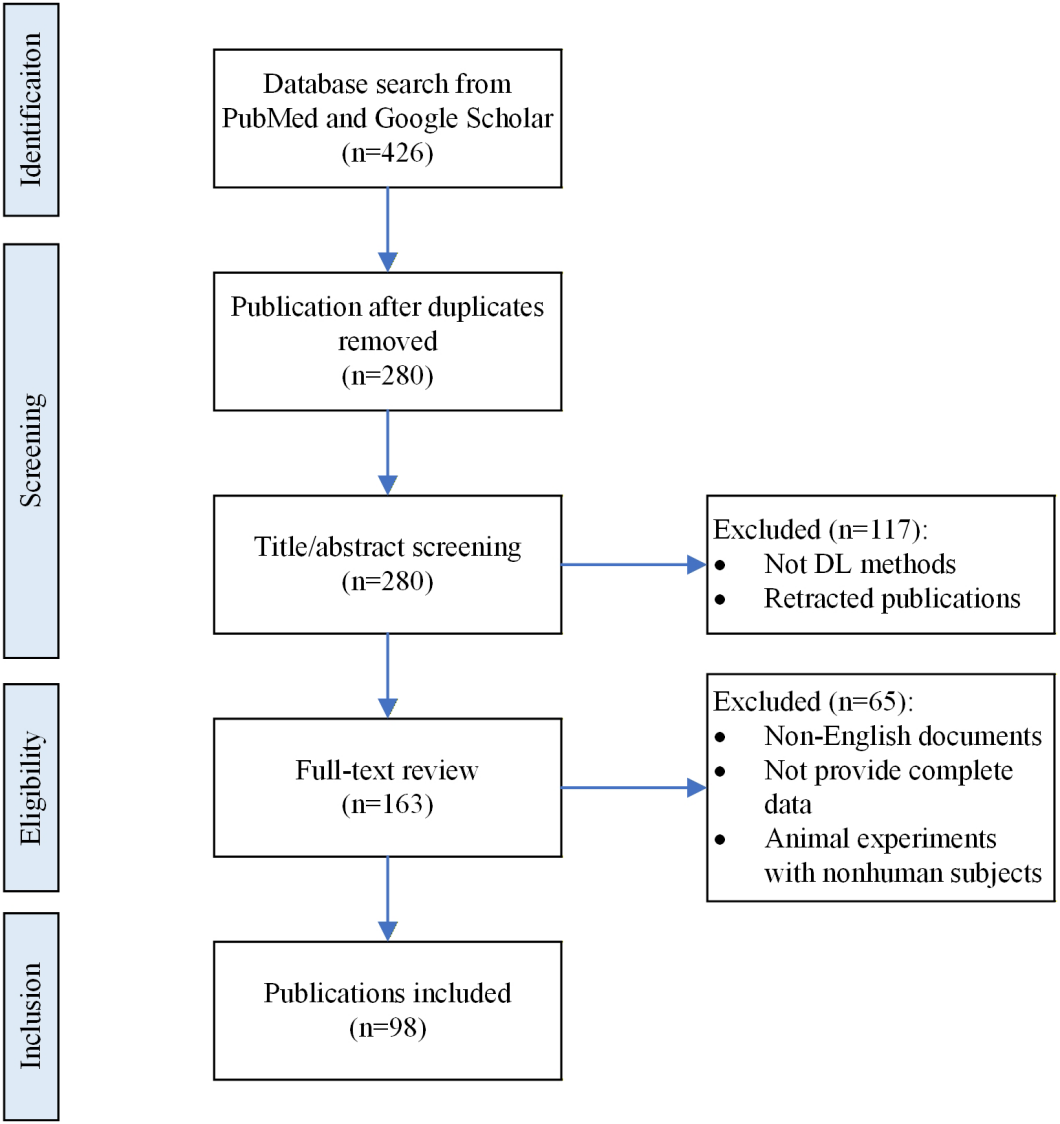
Selection criteria.

This study retrieved all the articles from the PubMed and Web of Science databases up to July 30, 2025. The search terms used were “artificial intelligence”, “ultrasound”, “kidney”, “renal”, and related terms. The literature screening process is shown in **Figure 2**. We followed the PRISMA guidelines and initially retrieved 426 articles from the PubMed and Google Scholar databases. After removing duplicates, 280 articles were retained for the screening stage. In the first round of screening on the basis of titles/abstracts, 117 articles were excluded for not meeting the criteria (exclusion reasons: articles that did not use the DL method/retracted articles). The remaining 163 articles were included in the full-text review stage. Following a detailed evaluation, 65 articles were excluded (for exclusion reasons: non-English literature/conference abstracts that did not provide complete data, reviews, etc./animal experiments with nonhuman subjects). Ultimately, 98 articles were included in this review.

## Technical foundation

The technical foundation of AI in the field of renal ultrasound relies mainly on core algorithm paradigms such as supervised learning, unsupervised learning, transfer learning, and multitask learning, which jointly drive a series of breakthroughs from image processing to advanced cognitive tasks (23, 24). Currently, supervised learning is the mainstream method, with its core lies in the use of expert-labeled data to train models to learn the mapping relationships between inputs and outputs (25, 26). In renal ultrasound image analysis, the following types of deep neural network architectures play a key role:

In 2015, the proposal of U-Net created an encoder-decoder architecture for medical image segmentation, which laid the foundation for kidney ultrasound segmentation. The subsequent variant (UNet++/nnU-Net) became the benchmark model for kidney segmentation. The outstanding local feature extraction ability of the convolutional neural network (CNN) significantly promotes the classification and segmentation performance of renal ultrasound images (27, 28). Studies have shown that CNNs perform well in tasks such as automatic segmentation and volume measurement of kidneys, especially in the automatic identification of hydronephrosis, where models based on CNNs can accurately capture the characteristic manifestations in images and effectively improve diagnostic consistency (29). ResNet alleviates the problem of gradient disappearance in deep networks by introducing residual connections, improving the recognition accuracy of complex anatomical structures (30). In renal ultrasound, structures such as ResNet50 have been proven to be able to more clearly identify renal boundaries and internal structures, indicating high value for CKD staging and renal tumor differentiation. DenseNet promotes feature reuse through dense connections, showing significant advantages in the case of limited training samples. In response to the characteristics of ultrasound images, attention U-Net introduces an attention mechanism to highlight key areas; U-Net++ improves the segmentation ability of small targets through nested skip connections (31); and nnU-Net becomes the benchmark model for multicenter research through an automatic configuration strategy (32). In 2017, the transformer model was born. Its self-attention mechanism solves the pain point of insufficient global feature capture by the CNN, which can effectively capture the global context relationship and significantly improve the modeling ability of the spatial relationship between the kidney and the surrounding tissues. Promote the upgrade of diagnosis from “local feature dependence” to “global relationship modeling” (20).

Unsupervised learning has unique value in scenarios with scarce labeled data in renal ultrasound. This method effectively expands the boundaries of supervised learning by mining the intrinsic patterns in unlabeled data. The main technical paths include 1) clustering analysis, which can be used to identify tissue characteristics of the renal cortex, medulla, etc., or distinguish different stages of chronic renal disease populations; 2) an autoencoder (AE), through an encoding-decoding structure to learn compact representations, whose derivative models, such as a denoising autoencoder (DAE), can improve image quality, and a variational autoencoder (VAE) can generate synthetic samples that conform to the real distribution to expand data; and 3) a generative adversarial network (GAN), through the game mechanism of the generator and discriminator, can synthesize realistic pathological images, alleviating the problem of insufficient samples (33). In 2020, cross-modal fusion technology has matured, and generative models such as CycleGAN have realized domain conversion from CT to ultrasound, overcoming the bottleneck of the scarcity of renal ultrasound labeling data and improving the segmentation accuracy in small sample scenes. These methods perform well in tasks such as feature learning, anomaly detection, and domain adaptation (34).

Transfer learning is another important way to alleviate the shortage of labeled data (35). Its core idea is to transfer the general features pretrained on large-scale source domains to the renal ultrasound task. Common strategies include the following: 1) feature extraction: fix the convolutional layer weights of the pretrained model as the feature extractor; and 2) fine-tuning: partially unlock the network layers and use the target data for iterative optimization. Practice has shown that this method can significantly reduce the dependence on the annotation scale, accelerate convergence, and improve the generalization performance. For example, by leveraging cross-modal transfer learning methods, accurate segmentation of renal ultrasound images can be achieved even under limited annotation conditions (36). Moreover, domain adaptation techniques further enhance the model’s robustness across different devices or centers by aligning the distributions between the source domain (annotated data) and the target domain (unannotated clinical data).

Multitask learning (MTL) efficiently utilizes annotation information by sharing underlying features and simultaneously optimizing multiple related tasks (such as segmentation, classification and volume measurement) 37. In renal ultrasound, MTL has three advantages: first, it improves overall performance by leveraging task correlations (such as using segmentation to assist volume estimation); second, it enhances generalization ability through knowledge transfer; and third, it reduces resource consumption for multi-model deployment. Since 2023: deep integration of large models and multimodal data. Through the combination of pretrained large models and cross-modal alignment technology, deep integration of ultrasound, CT, and genomic data has been achieved, which has improved the diagnostic accuracy of rare kidney disease and promoted the technology to expand from “single-task optimization” to “full-cycle kidney disease management” (34–36).

The three core algorithmic classes (CNNs vs. transformers vs. multimodal fusion) in the field of renal ultrasound DL differ significantly in terms of their technical characteristics, applicable scenarios and performance. The strengths, limitations and typical clinical suitability of these methods are shown in **Table 1**.

**Table 1:** Strengths, limitations and typical clinical suitability of the three core algorithmic classes.

Algorithms	Strengths	Limitations	Typical clinical suitability
CNNs	Strong local feature extraction ability, low training cost and high robustness	Global feature capture is insufficient and the effect of weak boundary segmentation is limited	Renal segmentation and volume measurement, lesion localization
Transformers	Self-attention mechanisms capture global spatial relationships and long-distance dependence	High computational complexity, sensitive to small samples, requires a large amount of pretraining data	Diagnosis of complex lesions, multiview image fusion
Multimodal fusion	Integrating complementary information to improve the comprehensiveness and accuracy of diagnosis	difficult to standardize the data and the design of the fusion mechanism is complex	Prediction of renal function and diagnosis of rare nephropathy

## The application of AI technology in renal ultrasound

### Renal segmentation and volume measurement

Renal ultrasound image segmentation and its derived volume measurement technology together constitute the core technical system of AI analysis of kidney diseases, and they show a close basic and extended relationship in clinical application (37–41). Segmentation is the premise of accurate measurement, and its accuracy directly determines the reliability of volume quantification, morphological evaluation and lesion localization (42–44). In turn, the clinical demand of volume measurement promotes the segmentation algorithm to be accurate and scenario iterative, forming a complete chain from morphological recognition to functional evaluation (45). Accurate segmentation and measurement have multiple synergistic meanings. First, three-dimensional reconstruction of the renal cortex, medulla and collecting system can be achieved by automatic segmentation, which can accurately calculate the total renal volume and cortical thickness and provide key quantitative indicators for the assessment of CKD progression and monitoring of renal allograft function. Studies have shown that renal cortical volume is significantly positively correlated with the glomerular filtration rate and that progressive volume reduction is an independent predictor of CKD progression (46). Second, the volume ratio of the renal pelvis to the renal parenchyma obtained by segmentation can provide an important basis for the timing of surgical intervention in patients with hydronephrosis, and the ratio is significantly related to the degree of renal function injury (47). Third, in clinical operations such as tumor ablation planning, accurate segmentation is the cornerstone of lesion localization and classification, whereas monitoring dynamic volume changes can indicate graft rejection or dysfunction early (48, 49).

Traditional methods (such as the threshold method, region growing method and active contour model) are limited by problems such as uneven gray levels in ultrasound images, noise interference and weak boundaries, which make it difficult to meet the requirements of boundary accuracy for volume measurement (50, 51). In recent years, deep learning technology has promoted the breakthrough progress of both methods. **Table 2** lists several representative studies on renal segmentation and volume measurements. In terms of segmentation algorithm innovation, Song et al. (52) and Guo et al. (53) used cross-modal data enhancement techniques (CycleGAN, CUT network) to effectively alleviate the problem of scarcity of labeled data through domain conversion from CT to ultrasound. For low-resolution images, Khan et al. (54) proposed that MLOU-Net introduces a deep supervised attention mechanism and hybrid loss function to achieve a 90.21% Dice coefficient on low-resolution renal ultrasound images. Alex et al. (55) designed the boundary feature enhancing network YSegNet combined with a long and short jump connection mechanism, and the Dice coefficient still reached 97% under the weak boundary challenge. Chen et al. (56) designed a multiscale feature fusion architecture (MSIP and MOS) to aggregate renal features of different scales, and the Dice index was 95.86%. Nipuna et al. (57) used 3D and multimodal fusion technology (3D U-Net fusion B-mode and power Doppler data) to achieve high-precision volume segmentation of the fetal kidney. Innovations in these segmentation techniques directly enable volumetric measurement applications. Jaidip M et al. (59) reported that the segmentation results of 3D ultrasound automatic measurements of ADPKD patients based on 2D U-Net and transfer learning were highly consistent with those of MRI (Dice=80%). The fast-unet++ proposed by Oghli et al. (60) can achieve high-precision segmentation (DSC>95%) of the sagittal and transverse planes and simultaneously predict multidimensional parameters such as renal length, width, thickness and volume. Kim et al. (61) developed a hybrid learning method of U-Net and an active contour model for the automatic calculation of renal volume in children, which was highly correlated with the CT measurement results (ICC = 0.925). Esser et al. (62) verified good interobserver agreement (ICC 0.83–0.94) via semiautomatic 3D ultrasound segmentation in the assessment of pediatric hydronephrosis.

**Table 2:** Application of DL technology in renal segmentation and volume measurements.

Author, Ref	Year	Algorithms	Data source	Size	Goals/Approach	Results
Yuxin Song et al. (52)	2022	CycleGAN	Single center, Public Dataset	391 images, 210 volumes	Cross-modal transfer learning	DSC=85.3%
Shuaizi Guo et al. (53)	2024	Seg-CycleGAN U-Net	Single center, Public Dataset	4883 images, 210 volumes	Improved model of CycleGAN	DSC=85.48%
Rashid Khan et al. (54)	2024	MLAU-Net	Single center, Public Dataset	44880 images	A deep supervised attention mechanism and a hybrid loss function are combined	DSC=90.21%
Deepthy Mary Alex et al. (55)	2022	YSegNet	Single center	700 images	Based on encoder, decoder and boundary extraction network	DSC=97%
Gongping Chen et al. (56)	2022	CNN	Single center	7350 images	New CNN	DSC=95.86%
Nipuna H et al. (57)	2021	3D U-Net, UNet++	Single center	780 volumes	3D U-Net/UNet++ combines 3D B-Mode and PD data	DSC=81%
Rashid Khan et al. (33)	2024	MLOU-Net	Single center	44880 images	Improve the deep neural network architecture and postprocessing methods	DSC=89.76%
Shuaizi Guo et al. (58)	2025	CUT, U-Net, CycleGAN	Single center, Public Dataset	4594 images, 210 volumes	A cross-modal data augmentation method based on CUT	DSC=77.19%
Jaidip M et al. (59)	2022	2D U-Net	Single center	22 patients	Automatic measurement of kidney volume by 3D ultrasound	DSC =80%
Mostafa Ghelich Oghli et al. (60)	2024	Mostafa Ghelich Oghli	3 Centers	744 images	Fast-Unet is optimized by nesting layers and deep supervision	DSC=95%
Dong-Wook Kim et al. (61)	2021	U-Net	Single center	331 children	U-Net + active profile	ICC=0.925
Michael Esser et al. (62)	2025	ICC, Dice	Single center	45 children, 48 volumes	Semiautomatic 3D ultrasound segmentation method	ICC=0.83~0.94

Existing studies have focused mostly on normal renal structure, and the generalization ability of tumors, cysts, severe hydronephrosis and other pathological conditions has not been fully verified. Moreover, these methods generally rely on single-center data and lack large-scale external datasets and prospective clinical trial verification across devices and institutions (45, 63). Future work should focus on developing robust segmentation algorithms for abnormal kidneys, constructing multi-disease and multicenter collaborative datasets, promoting the transformation of renal ultrasound AI systems from single-center research to real clinical scenarios, and verifying their feasibility and safety as routine diagnostic and treatment tools through multicenter clinical trials (64).

### Renal function prediction

Renal function prediction is a key step in the diagnosis and treatment of renal diseases (65, 66). Traditional methods rely mainly on biochemical indicators such as serum creatinine and urea nitrogen and use formulas such as CKD-EPI to estimate the glomerular filtration rate (GFR) (67, 68). However, these indicators are easily affected by muscle mass, diet and other factors and are less sensitive to early kidney injury (69). Traditional ultrasound interpretation is subjective and lacks quantitative analysis ability (70). In recent years, DL technology has significantly improved the objectivity and efficiency of renal function assessment through deep fusion of multimodal ultrasound data and clinical indicators to reduce human error (71, 72). Texture analysis can be used to extract functional information from images, overcome morphological limitations, and achieve quantitative descriptions of microstructures such as fibrosis and microangiopathy (73). In CKD, DL models can integrate multisource data such as electronic medical records, radiomics, and biomarkers to predict the risk of rapid progression of eGFR decline ≥5 mL/min/1.73 m² per year (74–76). In acute kidney injury (AKI), DL systems can identify high-risk patients before biochemical changes occur (77–80). **Table 3** lists several representative studies on renal function prediction.

**Table 3:** Application of DL technology in renal function prediction.

Author, ref	Year	Algorithms	Data source	Size	Goals/approach	Results
Ziman Chen et al. (81)	2023	Radiomics	Single center	160 patients	Combined with imaging features and clinical features	AUC=0.85
Han Yuan et al. (82)	2024	Combined model	Single center	332 patients	Viscoelastic imaging	AUC=0.91
Xinyue Huang et al. (83)	2025	Fisher	Single center	158 patients	Combined with clinical data, conventional ultrasound, shear wave elastography and vascular plane wave hypersensitivity imaging	Acc=84.7%
Yidan Tang et al. (84)	2024	Multimodal knowledge map	Single center	100 patients	Multimodal ultrasound	AUC=0.692
Ahmed M et al. (85)	2025	XAI-CKD	Single center	400 patients	Combined XAI-CKD model, BBFS feature selection, and SHAP interpretability analysis	AUC=1.0
Shuyuan Tian et al. (86)	2024	ResNet34	Single center	1049 patients	ResNet34 depth features and GLCM+HOG texture features were fused	AUC=0.781~0.931
Minyan Zhu et al. (87)	2024	SVM	Single center	117 patients	Combined with shear wave elastography, conventional ultrasound and color Doppler flow imaging	AUC=0.943
Fuzhe Ma et al. (65)	2020	HMANN	Single center	50 datasets	Fusion of features	Acc=97.5%
Fu Ying et al. (71)	2021	PCNN	Single center	20 patients	Ultrasound image enhancement algorithms	AUC=0.758
Chin-Chi Kuo et al. (88)	2019	ResNet-101, XGBoost	Single center	1299 patients	Deep fusion of ResNet-101 and XGBoost	AUC=0.904

Ziman Chen et al. (81) used radiomics to extract many quantitative features and combined them with clinical indicators to construct a prediction model, which achieved a noninvasive assessment of moderate to severe renal fibrosis (AUC = 0.85). Han Yuan et al. (82) used ultrasound viscoelastic imaging technology to assess renal function effectively and the degree of fibrosis via mechanical parameters such as the Emean and Vmean (AUC = 0.91). Xinyue Huang et al. (83) fused clinical data, conventional ultrasound, shear wave elastography, and plane wave hypersensitivity flow imaging to construct a Fisher discriminant model, which successfully distinguished different fibrosis grades (the highest accuracy was 84.7%). Yidan Tang et al. (84) integrated conventional ultrasound, contrast-enhanced ultrasound, and elastography to construct a multimodal ultrasound knowledge map and AI prediction model for the risk prediction of sepsis-related acute kidney injury. Ahmed M et al.’s XAI-CKD system (85), which is based on an extra tree classifier combined with SHAP interpretability analysis, achieved near-perfect performance (AUC = 1.0) in CKD classification. Shuyuan Tian et al. (86) integrated ResNet34 depth features and traditional texture features (GLCM+HOG) to achieve CKD diagnosis, especially in the G5 stage (AUC = 0.931). Minyan Zhu et al. (87) used an SVM to integrate various types of ultrasound image information and successfully predicted the degree of renal interstitial fibrosis (AUC = 0.943 when the IFTA > 50%). Fuzhe Ma (65) and Fu Ying (71) proposed the HMAN-based detection model and PCNN-based image enhancement algorithm, respectively, which improved the image quality and diagnostic reliability. Chin-Chi Kuo et al. (88) combined ResNet-101 and XGBoost to achieve automatic estimation of the eGFR and CKD grade (AUC = 0.904).

However, an examination of these representative studies reveals the core challenges facing the field (89–91). First, the generalizability of the models is generally questionable (92–94). For example, the near-perfect performance (AUC = 1.0) reported by Ahmed M et al. (85) is highly unusual in real medical data, strongly suggesting that the model may be overfitted on a specific dataset, and its cross-center applicability urgently needs to be verified. Second, the clinical translation of the technology faces a realistic bottleneck. For example, the multimodal fusion scheme of Xinyue Huang et al. (83) has improved performance, but its dependence on a variety of advanced imaging technologies is difficult to popularize in primary medical institutions, and the actual application cost is high. In addition, the limitations of research methods urgently need to be overcome. Although the pioneering work of Chin-Chi Kuo et al. (88) verified its technical feasibility, the limitations of its single-center design and lack of prospective validation are still common problems in many subsequent studies. In summary, although current research has made continuous breakthroughs in model performance, it is generally limited by key bottlenecks such as single-center data dependence, insufficient cross-center validation, and insufficient consideration of clinical applicability (95, 96).

### Renal disease diagnosis

Ultrasound imaging plays an irreplaceable role in the diagnosis of renal diseases (27). It is widely used in the assessment of renal morphology, screening of space-occupying lesions, diagnosis of hydronephrosis, monitoring after renal transplantation, and differentiation of cystic and solid lesions (97, 98). Especially in children, pregnant women and patients with renal insufficiency, ultrasound has become the preferred imaging method because of its safety (99). However, traditional renal ultrasound diagnosis also has obvious shortcomings: the results are highly dependent on the experience and skills of the operators, and there is strong subjectivity (100). It is not sensitive to early changes in renal function or slight structural changes (101). The ability of quantitative analysis is limited; for example, accurate assessment of renal fibrosis, diffuse lesions, or small hemodynamic changes is difficult. In addition, the low degree of standardization among different devices and scanning parameters also affects the comparability and repeatability of the results. DL technology has shown great potential in the diagnosis of renal diseases (102, 103). **Table 4** lists several representative studies on renal disease diagnosis.

**Table 4:** Application of DL technology in the diagnosis of renal diseases.

Author, ref	Year	Algorithms	Data source	Size	Goals/approach	Results
Miguel Molina-Moreno et al. (75)	2024	ResNet-50, Mask-RCNN	Single center	1985 images	Multitask convolutional neural network	AUC=0.819
Shi Yin et al. (104)	2020	CAKUT	Single center	157 children	Multi-instance deep learning method based on multi-view ultrasound images	AUC=0.961
Umar Islam Shi et al. (27)	2024	Novel DCNN	Public Dataset	1057 images	New two-path DCNN model	Acc=99.8%
Jinjin Hai et al. (105)	2021	CD-ConcatNet	Single center	76 patients	2D and 3D feature processing were fused to process multi-view ultrasound images	AUC=0.8667
S Sudharson et al. (98)	2020	ResNet-101+ ShuffleNet+ MobileNet-v2	Public Dataset	4940 images	Deep neural network ensemble model based on transfer learning	Acc=96.54%
Ming-Chin Tsai et al. (106)	2022	ResNet-50+ Transfer Learning	Single center	1599 images	Transfer learning model based on ResNet-50	AUC=0.959
Maosheng Xu et al. (22)	2025	Combined multimodal ultrasound	Single center	341 patients	Multimodal ultrasound combined diagnostic model	AUC=0.75

Miguel Molina-Moreno et al. (75) developed URI-CADS, an automatic system based on a multitask convolutional neural network, to realize the integrated analysis of kidney image segmentation and multi-pathological diagnosis, and its AUC reached 0.819 for multiple pathological diagnoses. Shi Yin et al. (104) proposed a multi-example deep learning framework to distinguish children with congenital anomalies of the kidney and urinary tract (CAKUT) effectively from those with unilateral hydronephrosis by clustering multi-view ultrasound image features. The AUC of the MIL model was as high as 0.961. Umar Islam et al. (27) designed a novel double-path convolutional neural network, which was significantly superior to classical models (such as VGG16 and ResNet50) in the detection of hydronephrosis, reaching 99.8% accuracy. Jinjin Hai et al. (105) integrated 2D and 3D convolutional structures to construct CD-ConcatNet to achieve fusion feature extraction and disease classification of multi-view renal ultrasound images (AUC = 0.8667). In addition, Sudharson et al. (98) achieved a high-precision four-classification task by integrating multiple pretrained models (ResNet-101, ShuffleNet, and MobileNet-v2) with an accuracy of 96.54%. Ming-Chin Tsai et al. (106) used transfer learning to optimize ResNet-50 to screen for children’s kidney abnormalities, and the AUC of the model was 0.959. Maosheng Xu et al. (22) combined two-dimensional ultrasound, color Doppler and shear wave elastography to construct a multimodal combined diagnostic model, and the AUC reached 0.75 in the classification of glomerular diseases in children.

These studies show the broad prospects of deep learning in improving the automation, quantification and multi-disease discrimination of renal ultrasound diagnosis (107, 108). However, the in-depth analysis identified several key issues of concern: first, the prudent evaluation of model performance. For example, the 99.8% accuracy reported by Umar Islam et al. (27) is extremely rare in medical image analysis and may reflect improper partitioning of the dataset or overfitting risk. Second, the clinical usefulness of complex models is questionable. Although the multitask system of Miguel Molina-Moreno et al. (75) has comprehensive functions, its stability under multicenter and different equipment conditions has not been verified. However, the multimodal method of Maosheng Xu et al. (22) has relatively limited performance (AUC = 0.75), suggesting that complex technology fusion may not improve performance. In addition, the study population was underrepresented. Most studies have focused on common conditions in children, and the applicability of these findings to complex abnormal renal structures (such as severe malformations and postoperative changes) remains to be investigated. In summary, these current studies are constantly innovating at the technical level, but there are still obvious deficiencies in the ability verification of model generalization and clinical practicality assessment (109).

## Challenges of DL technology in renal ultrasound applications

### Data-related challenges

#### Data quality and labeling

The data quality of renal ultrasound images directly affects the performance of DL models. Common noise, artifacts, and inconsistent resolution of ultrasound images lead to blurred renal structural boundaries and inapparent features, which affect the extraction of key information by the model. Guo et al. (58) demonstrated that a 30% reduction in the signal-to-noise ratio of ultrasound images can reduce the Dice coefficient of deep learning renal segmentation models by 8%-12%. High-quality labeling is the basis for training a reliable model, but renal ultrasound labeling faces many challenges (110). The unclear boundaries of the kidney and the lesion area lead to large differences in labeling between different doctors, and it takes 10–15 minutes for senior sonographers to label a single image (111). In addition, the inconsistency of labeling standards makes it difficult to integrate multicenter data and affects the generalizability of the model. Wu et al. (112) demonstrated that the inconsistency of observer labeling leads to a significant decrease in the average accuracy (AP) of a deep learning model. The AP50 value was 92.17% when the full labeling method was used, whereas the AP50 value increased to 98.57% when the local labeling method was used. To improve the quality of labeling, some studies have used enhanced data labeling strategies and automated pre-labeling techniques while evaluating labeling consistency through gradient mapping (105). These methods are helpful for improving the training effect of renal ultrasound AI models (113).

#### Insufficient data

In DL research on renal ultrasound, data scarcity, especially for rare diseases such as renal medullary cystic disease or hereditary nephritis, is the core challenge (114). Small samples can easily lead to overfitting and poor generalization ability of the model (115). Data heterogeneity in multiple centers further aggravates the problem of uneven data distribution. The DL model developed by Akbari et al. (64) showed high consistency (correlation coefficient >0.9) on single-center data, whereas the correlation coefficient decreased to approximately 0.8 when external multicenter validation was performed. To solve these problems, researchers have used data enhancement techniques (rotation, scaling, noise, etc.), which can improve the accuracy of small sample models by 5%-10% (116). Transfer learning can reduce the dependence on the amount of task-specific data by transferring generic features and still maintain high classification performance when the training samples are halved (117). In addition, cross-center data standardization and synthetic data generation, such as the diffusion model Med-DDPM, have also been explored to mitigate data scarcity and privacy issues. However, these methods still need more clinical validation to address potential limitations, such as algorithm transparency and data security.

### Technology-related challenges

#### Interpretability of the algorithm

At present, in renal ultrasound diagnosis, although complex DL algorithms such as deep neural networks have high accuracy, their “black box” characteristics make the decision-making basis difficult to understand, which severely restricts clinical trust and application (118). For example, models cannot explain why tumors with ill-defined boundaries are considered malignant, forcing physicians to rely on traditional pathological tests (119). Alderden et al. (120) used attention mechanisms to highlight key image areas, resulting in a 35% increase in physician trust. Feature visualization technology reveals decision logic by showing the texture, echo, and other features that the model focuses on (121). In addition, a lack of interpretability exacerbates ethical risks, making it difficult to assess potential biases of models against specific populations, such as different ages or genders (122). These challenges highlight the importance of developing interpretable artificial intelligence (XAI) methods. It is necessary to enhance transparency through visual interpretation, rule extraction and other techniques and establish a standardized ethical review framework to promote the safe application of AI in renal ultrasound diagnosis (123).

#### Robustness and generalization ability of the model

The robustness and generalizability of renal ultrasound AI models face multiple challenges, which are reflected in three main aspects: equipment differences, operator factors, and individual patient differences (124). The imaging principles and parameter settings of different ultrasound devices lead to differences in image feature distributions (125). For example, images from high-end ultrasound devices have high resolution and low noise, whereas images from primary hospitals may have obvious artifacts. The image quality was also affected by the operator’s scanning technique and section selection. The images of the same patient collected by different doctors may cause the Dice coefficient of the model segmentation results to fluctuate by 5%-7% (126). A small kidney size and incompletely developed structure in children and renal atrophy and fatty infiltration in elderly patients often lead to a 22% decrease in the performance of models trained for adults on children’s data (127).

### Clinical integration challenges

#### Integration with the clinical workflow

The core challenges of DL technology in the standardization of renal ultrasound data are the lack of standardization of multisource heterogeneous data and the differences in equipment models, imaging parameters, and operation specifications across different medical institutions, which limits the generalizability of AI models across institutions. The survey revealed that only 32% of the hospitals used unified scanning standards, which severely affected their clinical suitability (128). Števik et al. (129) explored the integration of AI into clinical workflows, but the need to manually upload images for analysis extended a single examination by 8–10 minutes, which did not meet clinical requirements for efficiency. This review revealed that AI-assisted diagnosis can significantly improve diagnostic accuracy. The diagnostic accuracy of AI-modified methods for complex renal diseases has increased by 21%, especially in the automatic detection of hydronephrosis and the classification of chronic renal disease, highlighting the advantages of standardization. Current technical bottlenecks include process interruptions caused by offline analysis modes and the lack of uniform image quality assessment standards, but real-time image analysis and computer-aided diagnosis systems enabled by convolutional neural networks have shown the potential to optimize workflows (130, 131). In the future, it is necessary to establish cross-platform data standards, develop embedded AI systems, and solve key problems such as algorithm interpretability and insufficient clinical validation to realize intelligent integration of the whole process from image acquisition to diagnostic reporting (132, 133).

#### Regulatory and ethical issues

Regulation and ethics are not to be ignored; Muralidharan et al. (134). reported that only 3.6% of FD-approved AI/IUI medical devices reported race/ethnicity, 99.1% did not provide socioeconomic data, and 81.6% did not report the age of the study subjects. The issue of data privacy is particularly prominent (135, 136). Because renal ultrasound images contain sensitive information, data sharing from multiple centers often conflicts with privacy regulations (137). In addition, ethical review should focus on algorithm fairness, including the evaluation of diagnostic differences in patients of different races and economic levels. The current solution emphasizes multiparty collaboration: the need to establish unified regulatory guidelines, improve data anonymization technology, develop a liability identification framework, and reduce algorithm bias through diverse dataset training is essential to promote the safe application of AI in renal ultrasound.

## Future prospects of DL technology in renal ultrasound

### Integration of emerging technologies

The integration of AI and multimodal imaging technology has significantly improved the diagnostic ability for renal diseases. With the anatomical details and functional information provided by CT and MRI, combined with the real-time advantages of ultrasound, the multimodal deep learning model can improve the diagnostic accuracy of renal tumor staging by 23% compared with that of a single ultrasound (138). The combination of molecular imaging technology and AI enables earlier molecular diagnosis, such as targeted contrast ultrasound molecular imaging, which can detect renal inflammation before renal dysfunction (139). The integration of wearable devices and Internet of Things technology has created a new mode of remote monitoring. Portable ultrasound devices can provide early warning through cloud-based AI analysis, and clinical trials have shown that acute exacerbation of chronic renal disease can be warned 3–5 days earlier (140). Given the limitations of single-center, single-modality and small samples, large medical models pretrained on large-scale multimodal data can extract shared representations of ultrasound-CT-MRI without massive labeling through cross-modal alignment and self-supervised learning. This approach significantly improves the ability to identify rare kidney diseases, such as hereditary nephritis, and alleviates the overfitting problem caused by scarce data. Edge computing technologies deploy lightweight AI models on portable devices to achieve point-of-care diagnosis. Federated learning achieved a diagnostic accuracy of 90.2% in the collaboration of 10 hospitals through the parameter sharing mechanism, which effectively solved the problem of data privacy (141). Combined with the federated learning and fine-tuning strategy, each center can share the basic model parameters while retaining local data, realizing multicenter coevolution and overcoming the bottleneck of single-center generalization. Augmented reality technology superimposes AI-processed tumor boundary information on the surgical field in real time, which improves the integrity rate of renal tumor resection by 18% (84). A large medical model pretrained on massive multimodal data shows strong adaptability and achieves 82% accuracy in the ultrasound diagnosis of rare renal diseases (142). The future large model will provide an interpretable basis for malignant diagnosis through visualization of the attention mechanism and chain-of-thought reasoning, transform black-box decisions into traceable clinical logic, and enhance the trust of doctors. These technical advances have promoted the rapid development of renal disease diagnosis, from morphological evaluation to functional, molecular, and real-time dynamic monitoring.

### Integrated intelligent diagnosis system

The ultimate goal of AI in the field of renal ultrasound is to develop an intelligent diagnostic system that can provide full-process, high-efficiency, and high-precision decision support for clinical practice through deep integration of a variety of AI functional modules and optimized human–computer interactions. The current research frontiers focus on building a one-stop diagnostic platform, deepening the application of personalized medicine, and promoting deep multimodal integration (143). The technical basis of the system is built on the framework of a multimodal large model fusion mechanism: unified representation learning is used to integrate ultrasound, CT, MRI and pathomics data, and a cross-attention module is used to achieve dynamic weighting of cross-modal features, which overcomes the limitations of traditional narrow AI, which only processes a single image. At the bottom of the platform, a lightweight real-time inference engine is deployed to compress the number of large model parameters to the scale that can be deployed on edge devices to meet the clinical needs of millisecond response. The core of the one-stop diagnostic platform seamlessly integrates the full chain of renal ultrasound AI applications, including real-time image quality assessment and standardized section guidance, automatic renal segmentation and volume measurement, dynamic prediction of renal function on the basis of image features and elastography parameters, intelligent identification and classification of common renal diseases, and automatic generation of structured diagnostic reports (144). The platform adopts the paradigm of large model pretraining + domain fine-tuning. First, self-supervised pretraining is carried out on millions of multicenter and multimodal data, and then efficient fine-tuning techniques such as LoRA are used to adapt local device parameters and population characteristics on the center-specific data to ensure cross-center generalization ability and localization accuracy. The platform uses a microservice architecture and workflow engine, allowing each AI module to call on demand, feed results to each other, and realize a closed loop of “scan, analysis, report” through a unified interface. Clinical verification shows that the integrated system can reduce the time of renal ultrasound examination and diagnosis by more than 40% and improve the consistency of diagnosis. By integrating the visualization research module of the attention mechanism, the closed loop can generate heatmaps in real time and overlay them on the original image, clearly label the decision basis of the model, and transform the black box into a transparent decision chain. The core of personalized medicine is the use of AI to mine multidimensional data of individual patients (such as dynamic changes in ultrasound imaging features, genetic background, serum/urine biomarker trajectories, comorbidities, and medication history in electronic health records) and the construction of patient-specific disease progression prediction models and treatment response models. For example, Bayesian deep learning models that combine trends in kidney texture features with genomic data can generate customized predictions of kidney decline trajectories for each CKD patient. The large model-based longitudinal dynamic fusion framework uses a temporal fusion transformer (temporal fusion transformer), which captures the imaging evolution of patients for months or even years, combined with time series data from electronic medical records, to realize dynamic risk warning and adaptive adjustment of treatment plans. Multimodal fusion is the technical cornerstone for achieving precision personalization. The future system will break through the current simple fusion of “ultrasound + clinical data” and evolve into deep heterogeneous data. Cross-image modality fusion uses AI to align and fuse the complementary information of renal ultrasound and CT/MRI/PET-CT. Radiomics and environment fusion integrate ultrasound radiomics features, serum/urine proteomics/metabolomics, and environmental exposure factors (116). The final multicenter, multimodal, and large-sample standardized platform aggregates multicenter data through federated learning. The basic model of 100 million parameters is trained to form a clinical decision-making center that can be transferred, interpreted and responded to in real time, and the fundamental challenge of the disconnect between traditional AI and clinical practice is completely solved.

### Multidisciplinary cooperation

The breakthrough of DL in the field of renal ultrasound requires deep interdisciplinary integration, integrating the expertise of computer scientists (algorithm design), imaging experts (image annotation and clinical relevance), nephrologists (diagnosis and treatment decision-making) and biomedical engineers (signal and software and hardware optimization) (145). Through joint discussion and clinical rotation to establish a common understanding, collaboration should run through the full cycle from clinical requirement definition, data collection, and model development to clinical verification to avoid technology being divorced from reality (146). This collaborative model can not only improve the reliability and interpretability of AI in the diagnosis of hydronephrosis and nephropathy but also optimize its clinical applicability so that resources can be focused on real bottleneck problems (147).

## Conclusions

DL has brought transformative advances to renal ultrasound, enhancing diagnostic accuracy, efficiency, and standardization across image segmentation, volumetry, disease diagnosis, and functional prediction while addressing traditional ultrasound limitations such as operator dependence and insufficient quantification—with performance comparable to that of professional physicians in renal structure recognition and lesion detection (148); however, critical research gaps, including uneven data quality, inadequate standardization/labeling, limited algorithm interpretability, poor cross-device generalization, clinical integration barriers, and incomplete regulatory/ethical frameworks, hinder its clinical translation, and accelerating this process requires targeted steps such as establishing unified data standards, developing explainable AI, deepening interdisciplinary collaboration, and refining regulatory guidelines, with future advancements in multimodal fusion, federated renal ultrasound systems, and medical large language models driving AI toward intelligent, personalized renal ultrasound systems that optimize the full workflow from image acquisition to clinical decision-making, ultimately enabling AI to become a core tool for precise renal disease diagnosis and treatment and supporting global kidney health management.
